# Feasibility of a randomised trial of Teaching Recovery Techniques (TRT) with refugee youth: results from a pilot of the Swedish UnaccomPanied yOuth Refugee Trial (SUPpORT)

**DOI:** 10.1186/s40814-022-00998-1

**Published:** 2022-02-14

**Authors:** Elisabet Rondung, Anna Leiler, Anna Sarkadi, Anna Bjärtå, Elin Lampa, Sandra Gupta Löfving, Rachel Calam, Brit Oppedal, Brooks Keeshin, Georgina Warner

**Affiliations:** 1 Department of Psychology and Social Work, Mid Sweden University, 831 25 Östersund, Sweden; 2 Child Health and Parenting (CHAP), Department of Public Health and Caring Sciences, Uppsala University, Box 564, BMC, Husargatan 3, 751 22 Uppsala, Sweden; 3 Division of Clinical Psychology, University of Manchester, Manchester, UK; 4 Division of Mental Health, Norwegian Institute of Public Health, Oslo, Norway; 5 Department of Pediatrics, University of Utah, Salt Lake City, UT USA

**Keywords:** Teaching recovery techniques, Post-traumatic stress disorder, Unaccompanied asylum-seeking and refugee minors, Randomised pilot trial, Feasibility

## Abstract

**Background:**

Although post-traumatic stress is prevalent among unaccompanied refugee minors (URM), there are few evidence-based psychological interventions for this group. Teaching Recovery Techniques (TRT) is a brief, manualised intervention for trauma-exposed youth, which has shown promising results in exploratory studies. The aim of the present study was to assess the feasibility of conducting a randomised controlled trial (RCT) evaluating the use of TRT among URM by investigating key uncertainties relating to recruitment, randomisation, intervention delivery and data collection.

**Methods:**

A 3-month long non-blinded internal randomised pilot trial with a parallel-group design assessed the feasibility of a planned nationwide multi-site RCT. URM with or without granted asylum were eligible if they were 14 to 20 years old, had arrived in Sweden within the last 5 years and had screened positive for symptoms of post-traumatic stress disorder (PTSD). Quantitative data were collected pre- and post-intervention, and 18 weeks after randomisation. On-site individual randomisation (1:1) followed directly after pre-intervention assessment. Participants allocated to the intervention were offered seven weekly group-based TRT sessions. Quantitative pilot outcomes were analysed using descriptive statistics. Qualitative information was gathered through on-site observations and follow-up dialogue with group facilitators. A process for Decision-making after Pilot and feasibility Trials (ADePT) was used to support systematic decision-making in moving forward with the trial.

**Results:**

Fifteen URM (mean age 17.73 years) with PTSD symptoms were recruited at two sites. Three of the youths were successfully randomised to either TRT or waitlist control (TRT *n* = 2, waitlist *n* = 1). Fourteen participants were offered TRT for ethical reasons, despite not being randomised. Six (43%) attended ≥ 4 of the seven sessions. Seventy-three percent of the participants completed at least two assessments, with a response rate of 53% at both post-intervention and follow-up.

**Conclusions:**

The findings demonstrated a need for amendments to the protocol, especially with regard to the procedures for recruitment and randomisation. Upon refinement of the study protocol and strategies, an adequately powered RCT was pursued, with data from this pilot study excluded.

**Trial registration:**

ISRCTN47820795, prospectively registered on 20 December 2018

## Key messages regarding feasibility

- In this pilot trial, uncertainties regarding the feasibility of conducting a randomised controlled trial in a population of unaccompanied refugee minors were investigated. The pilot trial specifically evaluated the feasibility of recruitment of study sites, intervention facilitators and youth, individual on-site randomisation, delivery of the group-based intervention Teaching Recovery Techniques (TRT) and data collection using an online survey.
- Key feasibility findings were that recruitment of sites, facilitators and youth was highly challenging and that individual on-site randomisation was not an optimal randomisation procedure. TRT could be delivered to youth according to plan, with acceptable attendance. Online data collection was feasible and appeared acceptable to the participants.
- Findings demonstrated a need for amendments to the trial protocol and strategies for a full-scale randomised controlled trial (RCT) to be feasible. Outreach materials and site recruitment procedures need to be adapted to new settings (i.e. schools) with increased support to remote sites. Individual randomisation will be replaced by cluster randomisation.

## Background

It is not surprising that seeking refuge from war is associated with vulnerability and mental health issues. The most vulnerable refugees appear to be unaccompanied refugee minors (URM) [1]. In 2015, 35,369 URMs sought asylum in Sweden. Most of them (86%) were boys and the majority came from Afghanistan, Syria, Somalia and Eritrea. The number of new applications decreased dramatically after 2015; however, many of the URM who arrived in 2015 still remain in the country [2]. The Swedish Migration Agency coordinates the housing for newly arrived URMs whom are often placed in residential care homes, which can be in any municipality. Each URM is provided a trustee (i.e. a legal guardian), safeguarding their rights and representing them in personal, legal and financial contexts.

In the years after 2015, the processing time for asylum applications increased and many refugees who had arrived in Sweden as minors turned 18. Their asylum applications were then to be treated as those of adults. To compensate for this, a new law (2017:353 [3]) was formed that allowed foreigners to receive a temporary residence permit whilst finishing upper secondary school. If they manage to find a job within six months of finishing upper secondary school, they are granted a permanent residence permit, but if not, they are deported.

Symptoms of post-traumatic stress disorder (PTSD) are reported by around a third to three-quarters of URM [4–6]. Research indicates that these symptoms persist over time [7–9] and could adversely affect URM integration into a new society by affecting their academic and employment prospects [10, 11]. Apart from PTSD, symptoms of anxiety and depression are also commonly reported (for a review, see von Werthern et al. 2019 [12]).

A recent meta-analysis assessing the effects of cognitive behavioural therapy (CBT) on symptoms of PTSD, depression and anxiety in child refugees identified 16 eligible trials, all of which point to an improvement in mental health following CBT, and the greatest reduction of symptoms in studies investigating PTSD [13]. Included studies specifically targeting URM were no exception [14–17]. However, despite the obvious need for mental health intervention in this group, and the positive impact of CBT demonstrated by Lawton and Spencer [13], refugees and asylum seekers in Europe commonly lack access to mental health services [18, 19]. The capacity of the mental health services treating PTSD is often insufficient [20] and most URM do not receive psychological treatment [21]. In order to optimise resource allocation and increase access to mental health care for these youth, a stepped care model of support featuring community-based group interventions could be developed. In Germany, several initiatives in this direction have taken place, such as in the MEHIRA project [22] and BETTER CARE [23]. To strengthen the evidence base for community-based interventions for this group, more evaluations of interventions like these are required.

Teaching Recovery Techniques (TRT) was developed by The Children and War Foundation, based in the United Kingdom and Norway [24, 25]. The brief, manualised intervention draws upon Trauma-focused cognitive behavioural therapy (TF-CBT) techniques to increase coping and promote recovery from PTSD in children aged 8 years and above who have experienced conflict or disaster. It was specifically designed to meet the needs of large groups of children in low-resource settings. High acceptability and large effect sizes for PTSD and depression symptom reduction have been reported in studies from Gaza [26] and after the tsunami in Thailand [27]. In a recent study from Baghdad, those with more severe PTSD-related symptoms demonstrated statistically significant improvement [28].

In Sweden, an exploratory study reported significant decreases in PTSD and depression among URM [17]. Over a fifth of participants recovered from their PTSD symptoms, whilst a third recovered from depressive symptoms.

The Swedish UnaccomPanied yOuth Refugee Trial (SUPpORT) project aims to further strengthen the evidence base of TRT among URM residing in Sweden [29]. Since there are only a few high-quality research studies evaluating interventions for newly arrived adolescent refugees [13, 15, 16, 30] a randomised controlled trial (RCT) was planned. However, engaging URM with traditional research methods has been identified as problematic [31] and it was anticipated that conducting an RCT with this group would be challenging. It is important not to overlook the feasibility of conducting RCTs with the target population. Some pioneering work has been conducted, see for example the studies on the intervention “Mein Weg” [15, 16], showing that brief group-based interventions can indeed be effective in reducing symptoms of PTSD among URMs. The meta-analysis by Lawton and Spencer [13] covers some feasibility aspects of delivering mental health interventions to refugee youth and children, such as the benefits of providing interventions at schools and of training lay personnel. However, in order to be able to conduct the methodologically rigorous primary studies that are asked for [13], there are further aspects to consider in relation to conducting RCTs with URM.

The aim of this pilot study was to assess the feasibility of conducting a full-scale RCT evaluating the effectiveness of TRT in improving URM self-reported mental health. After a 3-month pilot period, we thus sought to evaluate the major components of the trial as specified in the study protocol [29], giving high priority to issues of feasibility and acceptance. We specifically addressed uncertainties relating to the feasibility of:

- *Recruitment:* Number of sites and TRT facilitators engaged across the nation, and their geographical and organisational distribution. Rates of screening, eligibility and consent, along with any reported or identified reasons for non-consent.
- *Randomisation:* number of individuals successfully randomised and reasons for any randomisation failures.
- *Intervention delivery*: number of intervention groups initiated, number of TRT facilitators and participants per group, intervention attendance, any reported or identified reasons for non-attendance or failures in intervention delivery.
- *Data collection:* Retention rates, time needed to complete assessments, any reported or identified issues with regard to assessment procedures, measurements, translations, technical devices, transfer or storage of data and assessment acceptability. Indications of the appropriateness of main trial outcomes.

In the interest of the main trial, the overall aim of this pilot study was not only to identify and analyse any problems arising during the pilot period, but also to generate and assess possible solutions to these problems. In order to do so, A process for Decision-making after Pilot and feasibility Trials (ADePT) [32] was used. The aim was to inform necessary amendments in order to move on with the trial more efficiently. Although the Consolidated Standards of Reporting Trials (CONSORT) extension to randomised pilot and feasibility trials [33] does not perfectly apply to internal pilot and feasibility studies, the recommendations in this statement have been used when applicable.

## Methods

### Trial design

This study was planned as a 3-month long internal pilot (April–June 2019) of the SUPpORT trial [29], a parallel-group randomised controlled trial funded by the Kavli Trust (Grant ID A-321629). Ethical approval for the study was obtained from the Regional Ethical Review Board in Uppsala (Ref. 2018/382) on 28th November 2018 and the trial was prospectively registered at the ISRCTN Registry (Ref. ISRCTN47820795). The study protocol was developed by an interdisciplinary research team, with patient and public involvement (PPI) from a group of refugee advisors [34].

### Setting and recruitment

This pilot study was a joint effort by research teams at two Swedish universities, Uppsala University and the Mid Sweden University. Although we only had two sites with research team members (Uppsala and Östersund), the SUPpORT pilot was intended as a nationwide trial with multiple sites, targeting any sites with TRT-trained personnel. According to BRIS (Children’s Rights in Society), which is the main provider of TRT training in Sweden, about 350 people across Sweden have been trained to deliver TRT (personal e-mail communication with Somaya Ghanem, somaya.ghanem@bris.se, 15 September 2020). In the first phase of recruitment, community workers and project staff approached youth at places such as residential care homes, language cafés, non-governmental organizations and asylum health clinics. Different recruitment procedures were used at the two main sites. At one of the sites (Östersund), community workers in the youths’ ordinary network informed them briefly about the project and screened interested youth for eligibility. At the other site (Uppsala), the research team handed out leaflets, in the most common languages among refugees, and informed briefly about the study in several language cafés and non-governmental organizations. Additionally, school counsellors and personnel at asylum health care clinics were approached about referring eligible youth to the research team. All interested youth were invited to a first information meeting where they received more information about the study and were screened for eligibility. Youth were eligible to participate in the RCT if they were 14 to 20 years old, arrived in Sweden unaccompanied within the last 5 years (regardless of residence status) and screened positive on the Children’s Revised Impact of Event Scale (CRIES-8) [35] PTSD screening tool (≥ 17 points).

In the second phase of recruitment eligible youth were invited to ‘information and assessment meetings’ organised by project staff (with interpreters), where they received written and oral information about the study and gave their written informed consent to participate. For youth under 15 years of age, consent from their legal guardian was required. Once consent was obtained, the youth completed the pre-intervention assessment.

This was planned as an internal pilot of the main RCT. Rather than predetermining a specific number of participants in the pilot, we decided on a 3-month pilot period. This was documented beforehand in the trial protocol [29].

### Randomisation

Individual randomisation was planned to take place on site at the ‘information and assessment meetings’, on completion of the pre-intervention assessment. Consenting participants were to be randomised (1:1) into one of two groups [1]: TRT now (intervention) or (2) TRT in c.18 weeks (wait-list control). With an intended group size of 6-10 participants, block randomisation with random block sizes of 4 or 6 was considered a fair compromise to ensure relatively comparable group sizes. According to the initial protocol, 12 participants were considered enough to perform randomisation in order to reach the minimum required group sizes in each trial arm. Therefore, information meetings were to be performed until at least 12 participants had been recruited in each locality to enable individual randomisation that would result in at least two group formations. A research team member randomised participants using a password-protected website that hosted a computerised randomisation schedule, which was set up and maintained by a professional third party (www.sealedenvelope.com). The allocation to groups could not be influenced by project staff. Once allocated, neither project staff nor participants were blind to the assignment to groups.

### Intervention

Participants allocated to the intervention group were offered weekly group TRT sessions directly following randomisation, whilst participants in the waitlist-control group were offered the intervention at the end of the follow-up period. Participants who were not randomised were offered TRT for ethical reasons. Each TRT session lasted for 2 h, including a break. In its original format, the programme includes five youth sessions, including psychoeducation, affective modulation skills, cognitive coping and processing, trauma narrative, overcoming trauma reminders and future development. In addition to the five original youth sessions, a ‘getting to know each other session’ was offered at the beginning and a ‘follow-up’ session at the end of the intervention period. The youth were encouraged to nominate an adult they trusted to take part in the two ‘caregiver’ sessions included in the original TRT programme. According to the TRT manual, these sessions aim to introduce the TRT method to caregivers and instruct them in how to support the youth by maintaining routines and activities, and by listening and comforting when needed. During these sessions, caregivers are also informed about how to seek care if the youth needs additional help after TRT. Participants allocated to the wait-list control arm of the trial were able to access services as usual, then were offered TRT c.18 weeks after randomisation. An active control design was considered by the research group, but was not supported by PPI representatives.

### Data collection

Measurements of trial outcomes were administered pre-intervention (T1), after intervention delivery (T2) and c.18 weeks after randomisation/T1 (T3). Data were collected on tablets, using the Qualtrics platform for secure online data collection (Qualtrics; Provo, UT). Participants could use their language of choice (Swedish, English, Arabic, Dari, Farsi, Somali or Tigrinya) with interpreters present to give extra language support if needed. During the three assessment meetings, the youth were served food and drinks. To compensate for their time, they were also offered a shopping voucher valued at 100SEK at each assessment occasion.

Full details of outcomes in the main trial have been published in the trial protocol [29]. All outcome measures of the main trial were also assessed in this internal pilot. We thus collected data that, in the main trial, can be used to assess changes in youth self-reported mental health, specifically symptoms of PTSD (Children’s Revised Impact of Event Scale; CRIES-13 [35]), depression (Patient Health Questionnaire-9; PHQ-9 [36]) and anxiety (Generalized Anxiety Disorder-7; GAD-7 [37]). Secondary assessments included measures of self-efficacy (General Self-Efficacy Scale; GSE [38]) and well-being (Cantril Ladder [39]), both of which relate to the TRT programme theory of change. Basic demographic information and trauma history (Refugee Trauma History Checklist; RTHC [40]) were collected for all participants. Health-related quality of life (Child Health Utility 9D; CHU-9D [41]) and service consumption (Treatment Inventory of Costs in Patients with psychiatric disorders; TiC-P) Child and adolescent version [42]) were measured to inform an economic evaluation. A suicidality screening tool (Columbia-Suicide Severity Rating Scale (C-SSRS) Screen Version [43]) was utilised as part of a safety protocol for participants who indicated they have had thoughts they would be better off dead (scoring 1 or above, i.e. several days or more, in response to the item ‘Thoughts that you would be better off dead, or hurting yourself’, ninth item on PHQ-9) or ‘suffering’ on the Cantril Ladder (i.e. a score of 4 or below). Based on these criteria, we interviewed ten participants using the C-SSRS at pre-intervention, none at post-intervention, two at follow-up and four during the intervention. In some cases, the legal guardian was contacted and informed about the situation. None of the respondents were deemed to be at acute risk.

### Assessment of pilot study outcomes

Information regarding sites and TRT facilitators engaged across the nation was saved continuously. The number of individuals screened for eligibility, found eligible, consenting and randomised were logged during recruitment, as were the number of TRT groups, youth per group and TRT facilitators. At each TRT session, the facilitators filled out a fidelity checklist and an attendance list. The number of participants completing each assessment was saved automatically along with time logs for assessment completion. Descriptive statistics were used to consider the appropriateness of the trial outcomes.

Qualitative information about any reported reasons for non-consent or non-attendance, reasons for randomisation failures, issues relating to assessment procedures, data collection, intervention delivery, study logistics and multisite implementation were gathered narratively.

### Analysis of pilot study outcomes

Quantitative pilot outcomes were analysed using descriptive statistics. When applicable, rates and their corresponding 95% confidence interval (CI) were calculated. TRT session attendance and assessments on central trial outcomes (CRIES-13, PHQ-9, GAD-7, GSE and Cantril Ladder) were reported using the mean (with 95% CI and standard deviation) and median (with range). No statistical testing was undertaken as drawing inferences from the data was not an aim of this pilot study. Qualitative outcomes were described thematically.

The ADePT framework [32] was used to support systematic decision-making in moving forward with the trial. This process enables the generation and assessment of solutions to problems arising during pilot studies. First, the problems were classified into one of three categories; Type A are issues likely to be a problem only for the trial; Type B are issues likely to be a problem for both the trial and the real world; and Type C are issues likely to be a problem only for the real world. Next, solutions were generated by considering which aspects of the (i) intervention, (ii) trial design or (iii) context could be changed. Each solution was considered with regard to potential effectiveness and feasibility. Then, the most cost-effective single or multiple solutions were selected.

## Results

### Participants

Fifteen eligible URM, aged 16–20 years (*M* = 17.73, *SD* = 1.1, 95% CI 17.12-18.34), were recruited during the 3-month pilot phase of the trial, three by project staff in Uppsala and 12 by community workers in Östersund. Baseline demographics are presented in Table 1. Among the participants, two were female and 13 male. Five had come to Sweden from Afghanistan and 10 from Eritrea. All had lived in Sweden for less than 5 years. They completed the first assessment in Dari (2 participants), Tigrinya (9 participants) and Swedish (4 participants). On average, the participants had attended school for 7.33 years (95% CI 6.25-8.41, range 3-12). Due to a low number of youth randomised, we were unable to compare baseline demographics for the trial arms.

**Table 1:** Baseline demographics for participants in the SUPpORT pilot study (*n* = 15)

Demographic characteristics	*n*	(%)
Gender		
Female	2	(13.3)
Male	13	(86.7)
Nationality		
Afghanistan	5	(33.3)
Eritrea	10	(66.7)
Came to Sweden		
Alone	14	(93.3)
With family	1	(6.7)
Duration in Sweden		
Less than 1 year	5	(33.3)
1–2 years	4	(26.7)
2–3 years	3	(20.0)
3–4 years	3	(20.0)
Accommodation		
Family home	1	(6.7)
Home for care or housing	11	(73.3)
Lodger	3	(20.0)
Residence permit		
Temporary	11	(73.3)
Permanent	2	(13.3)
Waiting for interview	2	(13.3)

*Note:* Due to a low number of youth randomised, we were unable to compare baseline demographics for the trial arms

### Feasibility of recruitment

SUPpORT was intended as a nationwide trial with multiple sites, where TRT is delivered in a range of community settings. However, engaging new sites and recruiting already trained TRT facilitators was difficult. Despite researchers being in touch with several geographical sites, no sites besides the two research sites were successfully recruited during the pilot period. Neither could any previously trained TRT facilitators outside the research team be engaged. The primary reason for this was their lack of capacity, which related to a lack of dedicated funding to deliver TRT and/or a heavy workload of other tasks that prevented the allocation of work hours to deliver TRT. Another reason was staff turnover, which resulted in co-facilitators having left the organisation and sole facilitators not being able to deliver the intervention alone. In a few sites, the facilitators reported they were not currently in contact with URM that could receive the intervention. Instead, we arranged for new TRT facilitators to be trained and engaged TRT-trained research team members to facilitate groups. Due to the difficulties to engage new sites, only youth residing in the two regions where researchers were located (Uppsala and Östersund) were recruited during the 3-month pilot period.

**Fig. 1:**
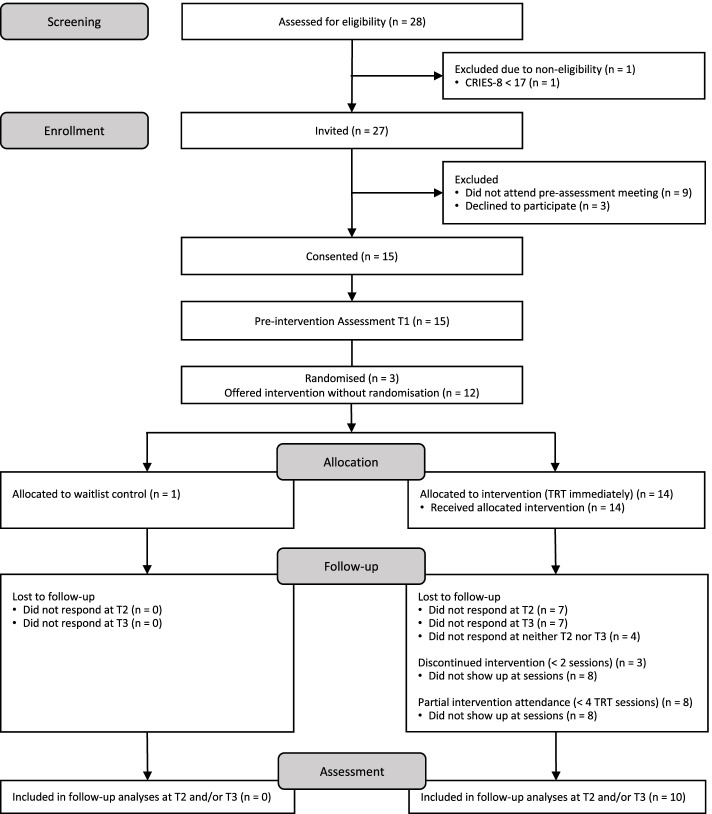
CONSORT flow diagram

In Fig. 1 we present a full CONSORT flow diagram of the participant numbers throughout the study. In the first phase of recruitment, a total of 28 youth were identified and screened for eligibility (16 in Uppsala and 12 in Östersund). All of the youth screened for eligibility during the pilot study period met the age and arrival status inclusion criteria, and 27 out of 28 met the PTSD symptom severity inclusion criteria (i.e. ≥ 17 on the CRIES-8). Hence, 96% (27/28, 95% CI 82%, 99%) of the individuals screened were eligible.

Of the 27 youth meeting all inclusion criteria (15 in Uppsala and 12 in Östersund), nine did not attend the ‘information and assessment meeting’ (the second phase of the recruitment) where the informed consent procedure took place despite multiple contact attempts by the research group and opportunity to participate at alternative times. Therefore, an immediate drop-out rate of 33% (95% CI 17%, 54%) from screening to consent was seen. At one of the sites (Uppsala), 6 out of 15 eligible youth (40%, 95% CI 16%, 68%) showed up. At this site, the ‘information and assessment meeting’ was held at a public place. Three out of the 6 who attended the meeting decided not to participate in the study due to personal reasons but agreed to participate in TRT groups, giving a consent rate of 20% (3/15, 95% CI 4%, 48%) at this site.

At the other site (Östersund), four out of 12 eligible youth (33%, 95% CI 10%, 65%) showed up at the first ‘information and assessment meeting’, when held in a public place. When a second meeting was arranged at the housing facility, where most of the eligible youth were living, all remaining youth showed up. This resulted in all 12 eligible youth at this site consenting to participate.

In total, the overall consent rate was 56% (15/27, 95% CI 35%, 76%). The initial recruitment target was 10 youth randomised per month. This was inclusive of an anticipated study drop-out rate of 41%, which was derived from a previous exploratory study of TRT delivery with URM in Sweden [17]. The recruitment target of 30 youth during the 3-month period was not met.

### Feasibility of randomisation

As shown in the CONSORT flow diagram (Fig. 1), only three out of fifteen participants were randomised, two to the intervention arm and one to waitlist control. This gave a randomisation rate of 20% (95% CI 4%, 48%). Ineligibility for randomisation was largely due to the interaction between the minimum number of youth required to deliver TRT (a group-based intervention), the on-site randomisation procedure and the participant-level randomisation. As the initial recommendations were that 12 youth should be randomised at once, we had too few participants at each of our three ‘information and assessment meetings’ (*n* = 3, *n* = 4 and *n* = 8). At the Östersund site, where the first ‘information and assessment meeting’ was arranged, this resulted in eligible youth consenting to participate in the study but not being randomised (*n* = 12). At the later ‘information and assessment meeting’ in Uppsala, the recommendations for randomisation were adapted, leading to three youth being randomised. The three youth who did not consent to be in the study but consented to be in TRT groups helped to form a reasonable group size for intervention delivery at this site. Once randomisation occurred, usage of the randomisation website worked smoothly.

### Feasibility of intervention delivery

Two intervention groups were arranged, one at each site (Uppsala and Östersund). In Uppsala, the group was led by two members of the research team. In this group, the two participants randomised to the intervention arm were included, together with three group members not included in the study.

In Östersund, many organisations were interested in delivering TRT. Thus, arrangements were made for 19 community workers and two psychology students to be trained. Of these, two community workers and the two students were active facilitators during the pilot period. Twelve participants were invited to the Östersund-based TRT group.

Of the 14 study participants who received TRT, six (43%, 95% CI 18%, 71%) attended four or more of the seven scheduled sessions. One participant attended all seven sessions, and two participants attended six out of seven sessions. On the other end, three participants only attended the first ‘getting to know each other session’. Besides that, the pattern of attendance was quite varied, with one participant attending two sessions, four participants attending three sessions, one attending four sessions and two attending five sessions.

Narrative reports from the TRT facilitators identified several challenges. It was difficult to find a good time and location for the sessions. Many participants had long school days and other activities after school. With regard to the location, a central but neutral location was chosen at both sites. However, with all participants at one site (Östersund) living at two residential care homes, the TRT facilitators tried to facilitate attendance by arranging all but the first session at the residential care home where most youth lived. Other challenges were related to the group composition, with ten boys and only two girls in one group, and with participants speaking two different languages. The varied pattern of attendance made progression between sessions difficult. At one site (Uppsala), this was solved with updates through text messages, emails and phone calls, with a summary of the session and written descriptions of the techniques. The facilitators also reported great difficulties in arranging the two caregiver sessions. In the end, no such sessions could be arranged at either site. Some of the youth were considered independent and had no stable adult in their lives. Others lived at residential care homes where practical reasons made the arrangement of caregiver sessions difficult. Limited flexibility due to non-compatible work schedules and non-flexible clinical duties posed another challenge on the facilitators, making it difficult to find time to plan and host meetings. It was also reported that it was difficult to find interpreters in a small town like Östersund. At times, this resulted in the need to reschedule TRT sessions. For the first two sessions in Uppsala a Swedish-Dari interpreter was present but a decision was taken, together with the participants, to continue with the remaining sessions in Swedish.

### Feasibility of data collection

At T1, 12 out of 15 participants completed the assessment online and three on paper (due to online questionnaires not being finalised). Four youth completed the questionnaires in Swedish (one online and three on paper), two in Dari and nine in Tigrinya. All post- (T2) and follow-up (T3) assessments were completed online, without complications.

On average, the online pre-intervention assessment took 61 min to complete (ranging from 35 to 84 min, *n* = 12). The follow-up surveys, which included fewer demographic questions (only those that may change during the study), took less time to complete, on average 38 min (range: 14–77, *n* = 8) at T2 and 47 min (range: 7–201, *n* = 8) at T3.

Observation reports from the assessment meetings indicated that the assessments, although translated, were difficult for some of the participants. Many asked the interpreter for help to understand questions and response alternatives. According to one of the interpreters, formal Tigrinya is complicated and difficult for many to understand. According to our on-site observations, most questions were raised regarding the demographic questions and the assessment of service consumption (TiC-P). Despite these difficulties, the assessments were generally well-accepted, and the youth worked intensively to complete them.

Based on the exploratory study of TRT with URM in Sweden [17], a retention rate of 59% was anticipated for the trial. In this pilot study, 53% (95% CI 27%, 79%) of the youth who completed the pre-intervention assessment at T1 also completed the post-intervention assessment at T2. The response rate of 53% was maintained at the T3 follow-up. However, the composition of the responding participants changed; 73% (95% CI 45%, 92%) of the participants completed at least two assessments (T1 and T2 or T3). Only 5 youth (33%, 95% CI 12%, 62%) completed all three data collection points (see Fig. 1).

At T1, mean levels of symptoms for all participants (*n* = 15) were for PTSD *M* = 32.13 (95% CI 25.64–38.63), for depression *M* = 12.73 (95% CI 9.59–15.87) and for anxiety *M* = 9.00 (6.00–12.00). At the same time point, mean ratings of wellbeing were *M* = 4.87 (95% CI 3.18–6.55) and self-efficacy *M* = 26.07 (95% CI 22.32–29.81). Five out of 15 of the participants scored below the cut-off on the CRIES-13 at T1.

Table 2 shows descriptive data from the three points of data collection for participants that received TRT (*n* = 14). It was hypothesised that reductions in mental health symptomatology and improvements in self-efficacy and wellbeing would be reported post-intervention. Pilot study data indicates this is the case, with the exception of self-efficacy for which the same level was maintained (see Table 2). No harm or unintended effects were reported.

**Table 2:** Summary of outcome measures at T1, T2 and T3 for participants receiving TRT, presented as mean scores (SD) above and median scores (range) below

	T1 (*n* = 14)^a^	T2 (*n* = 7)^a^	T3 (*n* = 7)^a^
**Primary outcome measures**			
CRIES-13	31.71 (12.06)	28.57 (15.67)	19.43 (9.78)
	33 (8–53)	31 (0–51)	22 (1–30)
PHQ-9	12.29 (5.61)	7.43 (5.25)	9.57 (7.18)
	12.50 (4–21)	10 (1–13)	7 (0–21)
GAD-7	8.57 (5.36)	5.14 (5.11)	5.14 (2.61)
	8.50 (0–19)	4 (0–14)	6 (0–8)
**Secondary outcome measures**			
GSE	26.64 (6.63)	26.14 (7.38)	24.71 (8.88)
	26.50 (10–36)	26 (17–36)	24 (10–38)
Cantril Ladder	5.07 (3.05)	7.43 (2.99)	8.57 (1.81)
	4 (1–10)	9 (2–10)	9 (5–10)

*Note:* ^a^Scores from participants randomised to waitlist control are excluded

## Discussion

In this pilot study, evaluating the feasibility of conducting a full-scale RCT investigating the effectiveness of TRT in improving URM self-reported mental health, quite a few challenges were encountered. Whilst some were directly applicable to this particular pilot trial (e.g. issues relating to randomisation and data collection), others may bare greater relevance when working with this group or this intervention in other settings (e.g. issues relating to recruitment, intervention delivery and multisite implementation). Importantly, our primary purpose in evaluating the feasibility was to inform how to proceed with this particular trial. The largely male sample from Afghanistan and Eritrea is fairly representative of URM in Sweden; yet, the specific context of this trial together with the low number of participants needs to be taken into consideration when generalising findings to other contexts or populations.

### Recruitment needs to be broad, customised and convenient to the youth

Many of the concerns were related to recruitment—of sites, TRT facilitators and youth. Although intended as a nationwide multi-site trial, we were not able to recruit any sites beyond our two research sites during the three-month pilot phase. Moreover, no previously trained TRT facilitators could be engaged to lead intervention groups. This low level of activity does not seem unique to this trial as communication from a network of TRT facilitators indicates that very few are currently active.

With only 28 youth screened and 15 consenting to participate, it became clear that recruitment efforts needed to be broadened, both geographically and across different arenas (e.g. into the school setting), for a full-scale trial to work out [44]. What can be done not to discourage potential participants in advance should also be considered. For instance, the RCT PPI panel highlighted how labelling potential participants as ‘refugees’ and referring to ‘mental health’ could be problematic. They also advised against individual randomisation, which they compared to ‘the lottery of seeking asylum’. Many eligible youths (*n* = 12 of 27) chose not to participate in the study, either actively by not giving their consent or passively by not showing up at the ‘information and assessment meeting’. In most cases, there was no opportunity to ask for their reasons not to come. It might have to do with non-acceptance of individual randomisation, but could just as well be related to mental health symptomatology affecting the youths’ ability to attend, experiences of stigma or the meeting taking place at an inconvenient time or location. A previous study of TRT with a sample similar to ours has, however, reported a high level of acceptability and appreciation for the groups [17]. As suggested in the theoretical framework of acceptability [45], there might be a reason to differentiate between prospective and retrospective acceptability. Hence, although acceptability of the intervention has been reported as high after participating in TRT [17], prospective acceptability of the intervention might not be equally high.

### TRT should be delivered in a format that facilitates intervention adherence

Once participants were included in the trial and offered TRT, a low level of adherence was anticipated in this group, given the instability of their life circumstances and the nature of their identified mental health symptomatology. This was also evident in the result. Although the number of TRT sessions is relatively low compared with other group interventions for trauma among refugee youth [46], weekly attendance can be challenging for youth experiencing trauma-related symptoms. This is especially true for those facing other challenging life circumstances, such as the asylum process. Low adherence might also relate to limited social and adult support or youth not finding the intervention to suit their needs and preferences. Given these circumstances, TRT facilitators have a great responsibility in facilitating attendance and progression throughout the intervention. Qualitative evaluation of the factors that have aided the implementation and maintenance of TRT delivery revealed that group facilitators should go to where the youth are, rather than expect them to come to where TRT is offered [47]. When URM are not residing together, one may need to consider other strategies, such as offering TRT in the school setting or online delivery of TRT. It is also important for group facilitators to stay in contact with participants in-between sessions; to remind in advance and give updates if a session is being missed. Intervention progression despite missed sessions was addressed at one site via remote updates, e.g. by text message, email or phone. Yet, brief in-person sessions with those who had missed the previous session before the next group meeting could also be considered. Adherence should continue to be monitored and the acceptability of the intervention, particularly new delivery formats, assessed both through consultation with PPI representatives and qualitative evaluation with study participants. Although contextual aspects such as policy updates and short-term funding initiatives have affected the implementation of TRT in Sweden [44], the unsuitability of the intervention should not be ruled out. Implementing another group intervention or low-level individual intervention to address the mental health needs of refugee minors could be explored if the implementation of TRT continues to be as challenging.

In TRT, caregiver sessions are viewed as a means to spread information about the intervention to important persons in the youths’ lives, so that they can provide additional support. Unfortunately, no caregiver sessions could be arranged during this pilot period. Experience of implementing TRT in Norway has also indicated challenges in conducting these sessions [48].

### Randomisation must be possible also when youth are few

With regard to the methodological features that are more specific to this particular study, the very low randomisation rate was the greatest concern. Despite the methodological benefits of individual randomisation, the large number of individuals needed to guarantee that each participant had a group to join made individual randomisation difficult. At the first ‘information and assessment meetings’, too few youths showed up to enable randomisation in accordance with the initial protocol. Once youth had been promised direct access to the intervention without randomisation, we chose not to change the procedures for this group for ethical reasons. When moving on to a full-scale trial, randomisation has to be possible even if fewer participants than 12 are available. The concerns raised against individual randomisation by the RCT PPI panel also need to be taken into consideration.

### Valid online data collection of suggested outcomes

Overall, the online data collection worked well, although the time needed to complete surveys varied greatly. Despite difficulties in understanding some of the questions, participants’ acceptance of the assessment method was generally high. The monetary incentives were potentially important in this regard. Retention was in line with what was expected. As stated in the protocol [29], linear mixed models and other statistical methods that make use of all available data thus seem like a reasonable choice for statistical analyses in the full-scale RCT.

The appropriateness of the outcome measure questions was considered beforehand based on the experience of the researchers in other child and adolescent mental health studies and via consultation with the RCT PPI representatives. As anticipated, participants showed high rates of mental health symptomatology and low levels of wellbeing, at both screening and pre-intervention assessment. Regarding the PTSD measure, five out of 15 participants scoring above the cut-off score on CRIES-13 at the screening did not reach the cut-off at the pre-intervention screening. Other screening tools for PTSD, such as the Child and Adolescent Trauma Screen [49], could be considered an alternative to CRIES. However, previous research has suggested the two versions of the scale (CRIES-13 and CRIES-8) to be efficient in classifying children with and without PTSD when using the cut-off scores of 30 and 17, respectively [35]. There are certainly explanations for the variation between the points of measure that can be traced to the psychometric properties of the scale. However, it could also be the case that daily life stressors affect the stability of measurement among this group.

Although the pilot study was not designed to assess the efficacy of the intervention, descriptive data on the main trial outcomes indicated a possible decrease in symptoms and increase in wellbeing from pre- to post- and follow-up assessments. This is in line with the hypotheses of the main trial [29] and indicates that the instruments used are likely to capture the anticipated reductions in mental ill-health and improvements in wellbeing. We should, however, be aware of the possibility that loss to follow-up could be skewing data, especially given the very small number of participants.

In line with our expectations, the current pilot data indicated relatively low levels of self-efficacy at pre-intervention compared with a Swedish cohort study [49]. There was no evident change post-intervention; however, T3 data indicated potential for change over time. Given general self-efficacy has previously been identified as a powerful predictor of posttraumatic recovery and still might mediate the effect of the intervention [50, 51], we chose to keep the variable for formal analysis in the full-scale trial.

### Applying the ADePT framework

**Fig. 2:**
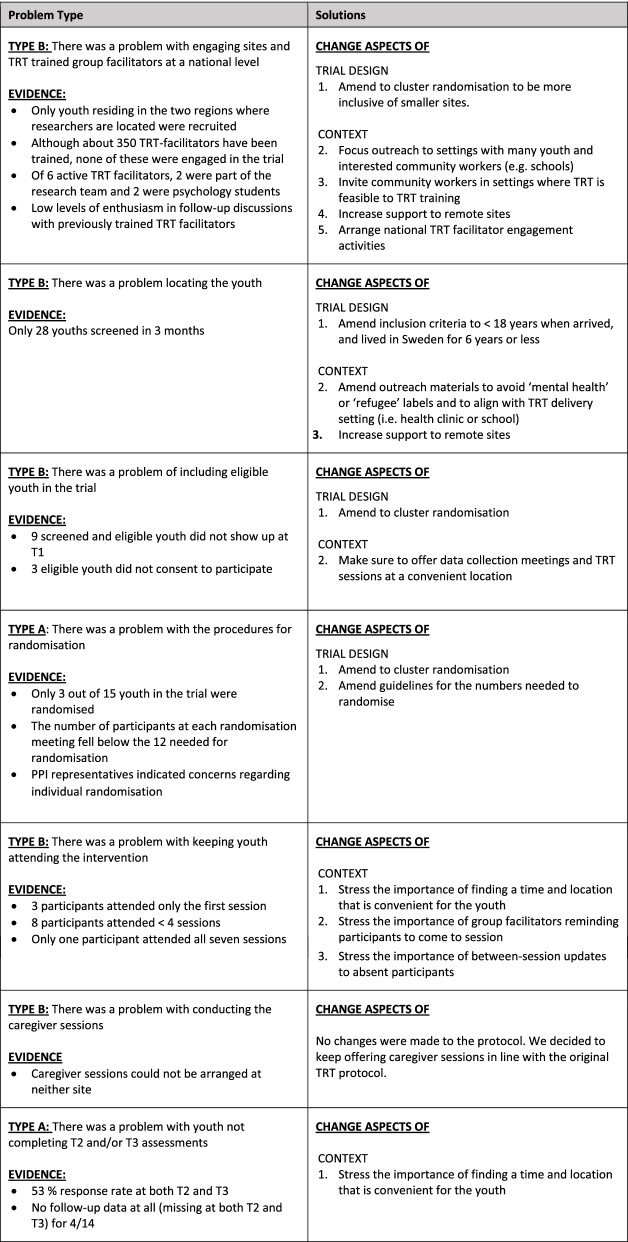
Identified problems and their solutions according to the ADePT framework

Although problematic, the challenges identified in this internal pilot were extremely valuable in informing the protocol for the main trial. By applying the ADePT framework, we identified seven problems during the pilot study period. Two of these were identified as Type A problems (likely to be a problem only for the trial), five were classified as Type B problems (likely to be a problem for both the trial and the real world) and none as a Type C problem (likely to be a problem only for the real world). A range of possible solutions were considered for each problem identified. The solutions were evaluated at team meetings, and when solutions were found likely to be feasible, efficient and cost-effective, amendments were made to the protocol. A list of the identified problems and actions taken to mitigate these are found in Fig. 2.

In moving on with the trial, recruitment will be the major concern. In order to recruit the predefined number of youth, outreach needs to be expanded to reach and include sites, TRT facilitators and youth across the nation. This will require great efforts in information and support. Strategies to monitor and enhance patient accrual and site enthusiasm, referred to by Weinberger et al. [52] as operational challenges specific to multi-site trials, will be of central importance. Thus, the research team has arranged workshops to collect good examples of how TRT can be implemented in various settings, exchanged ideas regarding effective recruitment strategies, set specific recruitment milestones, invited TRT facilitators across the nation to online gatherings, established step-by-step routines for how to best contact potential new sites, and increased telephone, video and on-site support to these sites. Yet, the COVID-19 pandemic has since complicated these processes and further affected recruitment efforts.

The very low randomisation rate identified an important issue in the trial design. Since individual randomisation both caused practical problems and was seen as problematic by the RCT PPI representatives, the decision was made to amend to cluster randomisation at the TRT group level. The target cluster average is 6 participants, based on TRT group size recommendations. However, randomisation can take place as long as a group size of at least 3 youth can be achieved. Groups can also include non-trial group participants. Not only is this likely to increase the numbers randomised, but also facilitate recruitment—especially at small sites. For larger sites, where it is possible that multiple TRT groups are generated over the course of the evaluation period, intra-cluster dependence will be explored as part of the analysis procedure. Since the technical procedures for randomisation worked well, these will be retained.

By applying the ADePT framework, the importance of facilitating for youth to come to assessments and intervention sessions was highly apparent. Encouragement for TRT facilitators to stay in touch with the youth in-between sessions to remind them to come, keep them involved in the process and make it easier to return to the group after being absent is also required.

In all, adopting the ADePT framework facilitated the identification and analysis of problems with the trial, as well as the generation and assessment of potential solutions (see Fig. 2 for an overview). Many important changes in study procedures can be implemented without amending the formal study protocol. However, in these regards, the protocol will be amended:

- Youth will be eligible if < 18 years of age when arriving in Sweden (the original protocol stated ages 14–20 at randomisation)
- Youth will be eligible if they have spent 6 years or less in Sweden (the original protocol stated 5 years or less)
- Individual randomisation will be amended to cluster randomisation allowing randomisation to take place as long as a group size of at least 3 youths can be achieved (groups can also include non-trial group participants).

With the aim of moving on to a full-scale RCT evaluating the effectiveness of TRT in its original mode of delivery, the suggested solutions and amendments all focus on strategies to enable face-to-face group delivery of TRT. However, our experiences during the pilot period also opened our eyes to the possibility of delivering TRT online, a direction we later elaborated further as a response to the COVID-19 pandemic [44]. Not only could this adaptation to the delivery format address the barriers to implementation encountered in this pilot trial, it could also facilitate the continuity of our efforts to provide trauma support to this population.

## Conclusions

Although some aspects of the trial were deemed feasible, a need for amendments to the protocol was evident, especially with regard to the procedures for recruitment and randomisation. With these amendments made, the decision was made not to retain data from pilot participants to the definitive trial. What was planned to be an internal pilot study thus ended up being an external pilot informing important updates of the protocol before moving on to a main trial. Upon refinement of the study protocol and strategies, an adequately powered RCT was pursued but has since been paused largely due to the COVID-19 pandemic. The findings from this pilot study may also bare relevance when working with this population or this intervention in other settings.

## Data Availability

The data are not publicly available due to ethical restrictions. The small data set contains information that could compromise the privacy of research participants. Requests to access the data should be directed to the corresponding author.

## Abbreviations

ADePT: A process for Decision-making after Pilot and feasibility Trials
BRIS: Barnens Rätt I Samhället (Children’s Rights in Society)
CHAP: Child Health and Parenting (research group)
CHU-9D: Child Health Utility 9D
CI: Confidence interval
CONSORT: Consolidated Standards of Reporting Trials
CRIES: Children’s Revised Impact of Event Scale
C-SSRS: Columbia-Suicide Severity Rating Scale
GAD-7: Generalized Anxiety Disorder-7
GSE: General Self-Efficacy Scale
PHQ-9: Patient Health Questionnaire-9
PPI: Patient and public involvement
PTSD: Post-traumatic stress disorder
RCT: Randomised controlled trial
RTHC: Refugee Trauma History Checklist
SUPpORT: Swedish UnaccomPanied yOuth Refugee Trial (project titel)
TF-CBT: Trauma-focused cognitive behavioural therapy
TiC-P: Treatment Inventory of Costs in Patients with psychiatric disorders
TRT: Teaching Recovery Techniques
URM: Unaccompanied refugee minors
